# Zinc-Regulated DNA Binding of the Yeast Zap1 Zinc-Responsive Activator

**DOI:** 10.1371/journal.pone.0022535

**Published:** 2011-07-22

**Authors:** Avery G. Frey, Amanda J. Bird, Marguerite V. Evans-Galea, Elizabeth Blankman, Dennis R. Winge, David J. Eide

**Affiliations:** 1 Department of Nutritional Sciences, University of Wisconsin-Madison, Madison, Wisconsin, United States of America; 2 Department of Biochemistry, University of Utah Health Sciences Center, Salt Lake City, Utah, United States of America; University of Cambridge, United Kingdom

## Abstract

The Zap1 transcription factor of *Saccharomyces cerevisiae* plays a central role in zinc homeostasis by controlling the expression of genes involved in zinc metabolism. Zap1 is active in zinc-limited cells and repressed in replete cells. At the transcriptional level, Zap1 controls its own expression via positive autoregulation. In addition, Zap1's two activation domains are regulated independently of each other by zinc binding directly to those regions and repressing activation function. In this report, we show that Zap1 DNA binding is also inhibited by zinc. DMS footprinting showed that Zap1 target gene promoter occupancy is regulated with or without transcriptional autoregulation. These results were confirmed using chromatin immunoprecipitation. Zinc regulation of DNA binding activity mapped to the DNA binding domain indicating other parts of Zap1 are unnecessary for this control. Overexpression of Zap1 overrode DNA binding regulation and resulted in constitutive promoter occupancy. Under these conditions of constitutive binding, both the zinc dose response of Zap1 activity and cellular zinc accumulation were altered suggesting the importance of DNA binding control to zinc homeostasis. Thus, our results indicated that zinc regulates Zap1 activity post-translationally via three independent mechanisms, all of which contribute to the overall zinc responsiveness of Zap1.

## Introduction

The activity of transcriptional regulatory proteins can be regulated by a variety of mechanisms. These include the control of transcription factor expression, stability, subcellular localization, DNA binding activity, and activation domain function. While the activity of many transcription factors is regulated by only a single mechanism, some are regulated at multiple levels. Multiple levels of regulating a single transcription factor allow that factor to respond to different signals. For example, the C/EBP transcription factors are regulated at transcriptional, translational, and post-translational levels [1]. These different mechanisms allow C/EBP target gene expression to be controlled by cell differentiation, hormone levels, MAP kinase cascades, and calcium signaling.

Another advantage of multiple regulatory mechanisms controlling the activity of a transcription factor is the combined effects each mechanism can have on the response of that factor to a single stimulus. For example, the yeast Pho4 transcription factor is regulated in response to phosphate status by phosphorylation at four different sites in the protein [2]. Phosphorylation at one site inhibits the interaction of Pho4 with its partner protein, Pho2, thus decreasing activation function. Phosphorylation at two other sites in Pho4 promotes nuclear export of the protein while phosphorylation at a fourth site inhibits Pho4 nuclear import. These different mechanisms of regulation are all required for full repression of Pho4 activity by phosphate.

In this report, we address the multiple levels of regulation that control the yeast Zap1 protein in response to zinc status. Zinc is an essential nutrient for all organisms because of its many functions as a structural and catalytic cofactor. Zinc is also potentially toxic to cells when accumulated in high amounts. The essential but potentially toxic nature of this metal necessitates precise homeostatic control mechanisms. In *E. coli*, zinc homeostasis is accomplished largely through the transcriptional control of zinc uptake and efflux transporters [3], [4]. Studies of the regulatory zinc sensors that control expression of these transporters suggest that *E. coli* cells strive to maintain essentially no free zinc in their cytosol [5]. Similarly, in eukaryotic cells, cytosolic free zinc levels are estimated to be at or below nanomolar levels under steady state growth conditions [6], [7], [8].

We know much about zinc homeostasis in eukaryotes from studies of the yeast *Saccharomyces cerevisiae*. The Zap1 transcriptional activator is a central player in the adaptation of these cells to zinc-limiting conditions. Zap1 activates the expression of as many as 80 genes [9], [10] and also represses the expression of other targets through various mechanisms [11], [12], [13]. Genes induced by Zap1 encode proteins such as the plasma membrane zinc transporters Zrt1, Zrt2, and Fet4, the vacuolar zinc transporters Zrt3 and Zrc1, and other proteins involved in adaptation to zinc deficiency [9].

Zap1 is an 880-residue protein containing seven C_2_H_2_ zinc fingers (Znf). Znf3-7 are located at the C-terminus (residues 705–880) and comprise the DNA-binding domain. This domain is responsible for specific recognition of Zinc Response Elements, or ZREs, found in one or more copies in Zap1's target promoters [14]. The consensus ZRE sequence is 5′-ACCTTNAAGGT-3′ and we have shown previously that Zap1 binds to this palindromic sequence as a monomer and not as a dimer [15].

Zap1's two activation domains, AD1 and AD2, are responsible for increasing gene expression in response to low zinc. AD1 lies between residues 207 and 402 and is embedded within a larger region (residues 182–502) termed the Zinc-Responsive Domain of AD1 (ZRD^AD1^) that is required for controlling AD1 function [16]. Zap1 AD2 maps between residues 611 and 641, which are the endpoints of the Znf2 zinc finger [17]. The ZRD for AD2 (ZRD^AD2^) includes both Znf1 and Znf2. Studies to date indicate that zinc regulates AD1 and AD2 directly by binding to ligand residues in their respective ZRDs to repress activation domain function [16], [17].

While regulation of AD1 and AD2 clearly plays an important role in Zap1 zinc responsiveness, other levels of control may also contribute. For example, at the transcriptional level, Zap1 induces its own expression in low zinc via positive autoregulation [18]. The contribution of autoregulation to Zap1's overall zinc responsiveness is unknown. In addition, activity of a fusion protein in which the Zap1 DNA binding domain (residues 687–880) alone was attached to the Gal4 activation domain (GAD-Zap1^DBD^) was zinc regulated [19]. This result suggested that zinc controls Zap1 DNA binding. However, equally viable alternative mechanisms of zinc regulation of this fusion protein included control of Zap1 nuclear localization and recruitment of co-repressors (e.g. histone deacetylases) to promoters by this domain. In this report, we have tested these hypotheses and found that DNA binding activity is indeed regulated by zinc. Furthermore, we present evidence that this control is a critical component of Zap1's zinc responsiveness.

## Results

### Some zinc regulation is mediated by the Zap1 DNA binding domain *in vivo*

**Figure 1:**
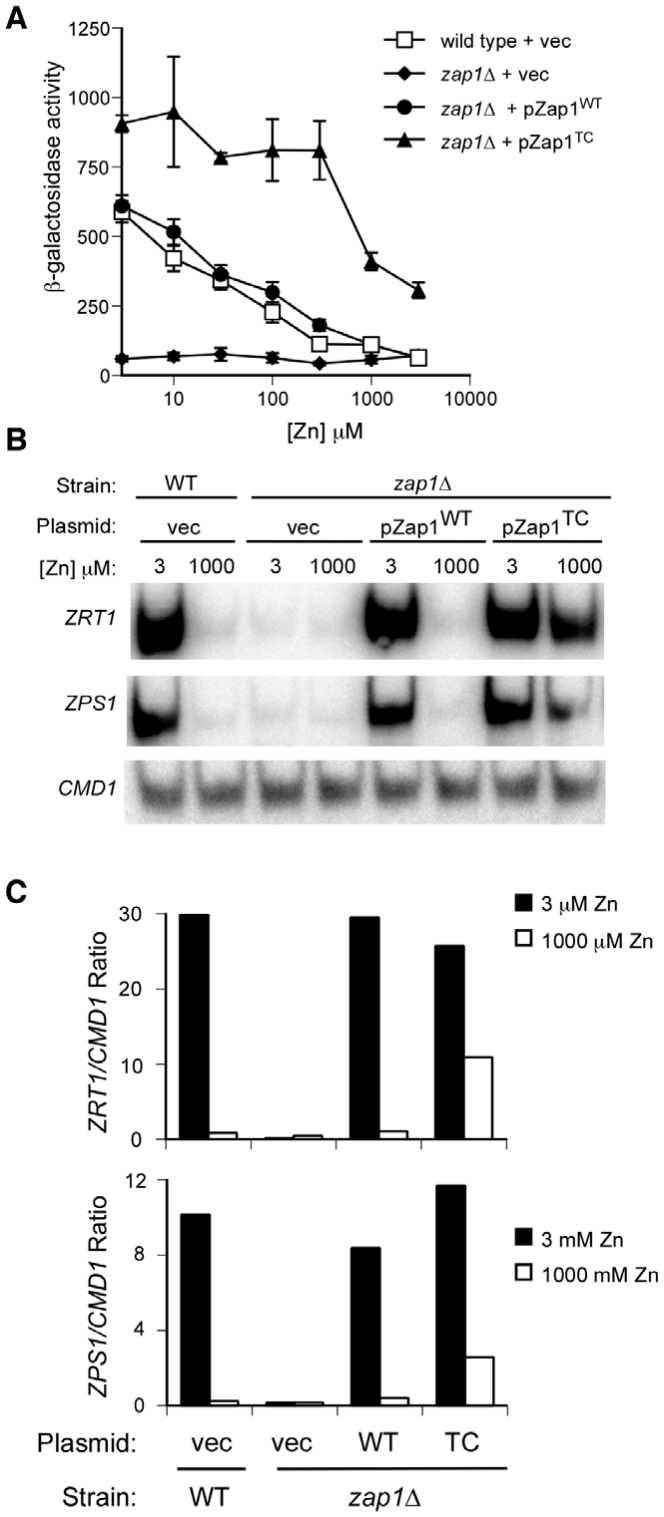
Possible zinc regulation of the Zap1 DNA binding domain *in vivo*. **A**) Wild-type (DY1457) or *zap1Δ* (ZHY6) cells transformed with either pZap1^WT^, pZap1^TC^, or the pYef2 vector and the pGI-1 *ZRT1-lacZ* reporter were grown to exponential phase in LZM supplemented with the indicated concentration of ZnCl_2_ prior to analysis of β-galactosidase activity. Zap1^TC^ has mutations in the zinc-responsive domains of Zap1's two activation domains rendering those domains constitutive. *ZRT1* encodes a plasma membrane zinc uptake transporter and is a Zap1 target gene. Values plotted are the means of three replicate cultures and the error bars indicate ±1 S.D. **B**) Total RNA was extracted from the cells described in panel A grown in LZM supplemented with 3 µM or 1000 µM ZnCl_2_ and S1 nuclease assays were performed to determine mRNA levels of *ZRT1* and *ZPS1*, another Zap1 target gene that encodes a secreted protein of unknown function. *CMD1* was used as a loading control. **C**) Graphical representation of results obtained in panel B.

In wild-type cells that express Zap1 from its own promoter, a Zap1-regulated *ZRT1-lacZ* reporter is highly induced by zinc limitation and expressed at decreasing levels as the concentration of zinc in the medium is increased (**Figure 1A**). *zap1Δ* cells expressing wild-type Zap1 at a low level from the *GAL1* promoter (pZap1^WT^) showed a similar response. Although this promoter is highly induced by galactose, we grew these cells in glucose where the level of Zap1 expression was similar to the level observed in zinc-replete wild-type cells expressing Zap1 from its own promoter [20] and much less than in galactose-grown cells (see below). To assess whether zinc-regulated Zap1 DNA binding may contribute to this response, we assayed *zap1Δ* cells expressing a mutant form of the protein (Zap1^TC^) in which AD1 and AD2 function is made constitutive by mutating regulatory residues required for the zinc responsiveness of those domains [16] (**Figure 1A**). As previously shown, this mutant allele showed little zinc response under moderate conditions (i.e. 3–300 µM Zn). However, when zinc was added at higher levels (i.e. 1000 and 3000 µM), reporter expression was reduced to approximately 30% of maximal levels. These effects were confirmed when mRNA levels of *ZRT1* and *ZPS1*, two chromosomal targets of Zap1, were assayed under low and high zinc conditions (**Figure 1B, C**). It should be noted that LZM, the medium used in these and subsequent experiments, contains 1 mM EDTA and 20 mM citrate as metal buffers to limit zinc availability. Therefore, even at high total zinc concentrations (e.g. 1000 or 3000 µM) the level of free Zn^2+^ in the medium available to cells is less than what is found in standard yeast culture media (i.e. YPD or SD) [21]. *CMD1*, encoding calmodulin, was used as a negative control and its levels were not altered by zinc status.

### ZRE occupancy is regulated by zinc status *in vivo*

**Figure 2:**
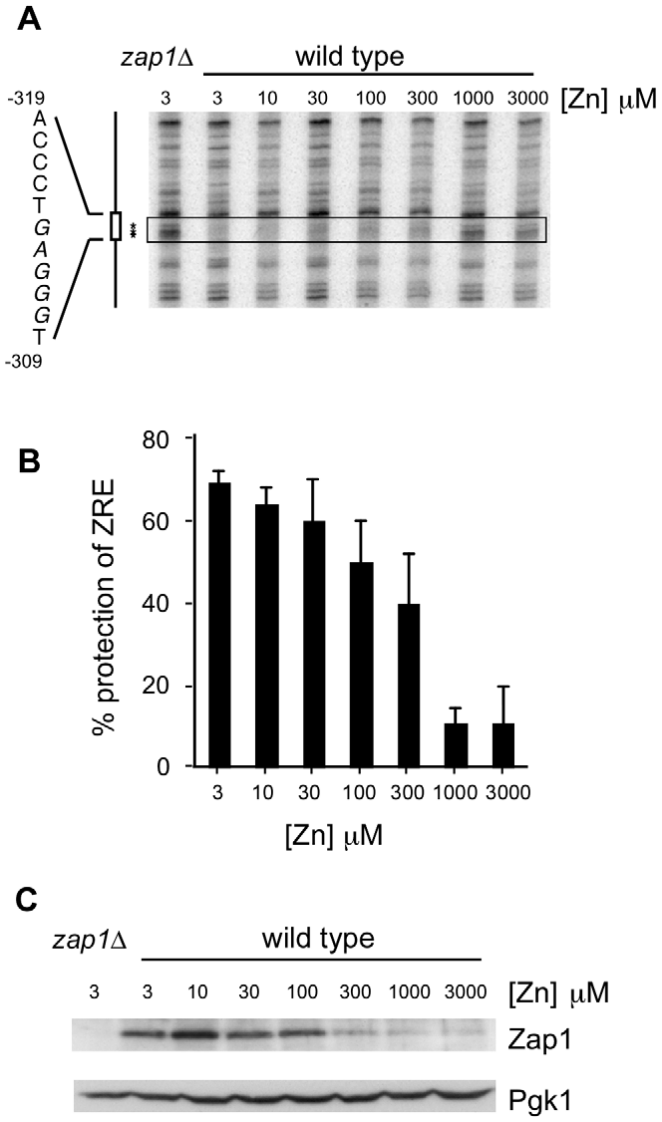
ZRE occupancy by Zap1 is altered by zinc status *in vivo*. **A**) Wild-type (DY1457) cells were grown to exponential phase in LZM supplemented with the indicated concentration of ZnCl_2_ and analyzed by *in vivo* DMS footprinting on the *ZRT1* promoter. A sample from a ZHY6 *zap1Δ* mutant grown in LZM+3 µM ZnCl_2_ is shown for comparison. The position of the ZRE was determined using a DNA sequencing ladder (not shown) and is indicated by the *box*. The sequence of the ZRE is shown with the distance of those bases from the ATG start codon; the purines that are sensitive to DMS methylation are *italicized*. The ZRE bands used to quantify protection are marked by *asterisks* and *boxed*. **B**) The experiment shown in Figure 2A was repeated a total of six times and quantified as described in the Materials and Methods. The mean percent protection levels are shown and the error bars indicate 1 S.D. **C**) Immunoblot analysis of Zap1 and Pgk1 levels in the same cells as in Figure 2A. Total protein extracts were prepared from aliquots of cells harvested prior to DMS treatment.

The observation that the Zap1^TC^ protein and the GAD-Zap1^DBD^ fusion [19] retain substantial zinc regulation suggested the possibility that Zap1 DNA binding activity was controlled by zinc. To test this hypothesis, we first used *in vivo* dimethyl sulfate (DMS) footprinting to examine occupancy of a Zap1 binding site (ZRE1) in the *ZRT1* promoter [22]. In a *zap1Δ* mutant grown in zinc-limiting (**Figure 2A**) or replete (data not shown) conditions, a cluster of purine nucleotides within the ZRE was readily accessible to methylation by DMS. In zinc-limited wild-type cells where Zap1-activated transcription is high, these ZRE residues were largely protected from methylation consistent with Zap1 occupying the site *in vivo*. With increasing zinc levels, ZRE protection decreased suggesting loss of Zap1 binding. To quantify the effects of zinc on ZRE occupancy, the experiment shown in **Figure 2A** was repeated six times, the band intensities were measured, and the results are plotted in **Figure 2B**. Fractional ZRE protection in zinc-limited wild-type cells was estimated to be 68% (setting the methylation level observed in a *zap1Δ* mutant at 0% protection) (see Materials and Methods). Less than 100% methylation protection may be observed if there is incomplete ZRE occupancy in low zinc or only partial protection by bound protein. In zinc-replete (e.g. LZM+1000 µM zinc) wild-type cells, methylation protection dropped to ∼10% indicating a dramatic loss of Zap1 binding.

Zap1 controls its own transcription via positive autoregulation. Therefore, the effects of zinc on ZRE occupancy in wild-type cells could result solely from changes in Zap1 protein level. To assess this possibility, we first determined the level of Zap1 protein expressed under these conditions by immunoblot analysis (**Figure 2C**). Consistent with the effects previously observed for *ZAP1* mRNA [10], [18], Zap1 protein levels were highest in zinc-limited cells and decreased as the zinc concentration of the medium was raised. Little effect of zinc status on the level of a control protein, the Pgk1 3-phosphoglycerate kinase, was observed. Having shown previously that zinc does not trigger degradation of Zap1 protein [16], these data indicated that *ZAP1* transcriptional autoregulation does indeed cause corresponding changes in Zap1 protein level. Therefore, the apparent loss of *ZRT1* ZRE occupancy observed in wild-type cells grown in high zinc could be due to decreased Zap1 DNA binding activity and/or decreased Zap1 protein level resulting from transcriptional autoregulation.

### Zinc regulation of ZRE occupancy does not require Zap1 transcriptional autoregulation

**Figure 3:**
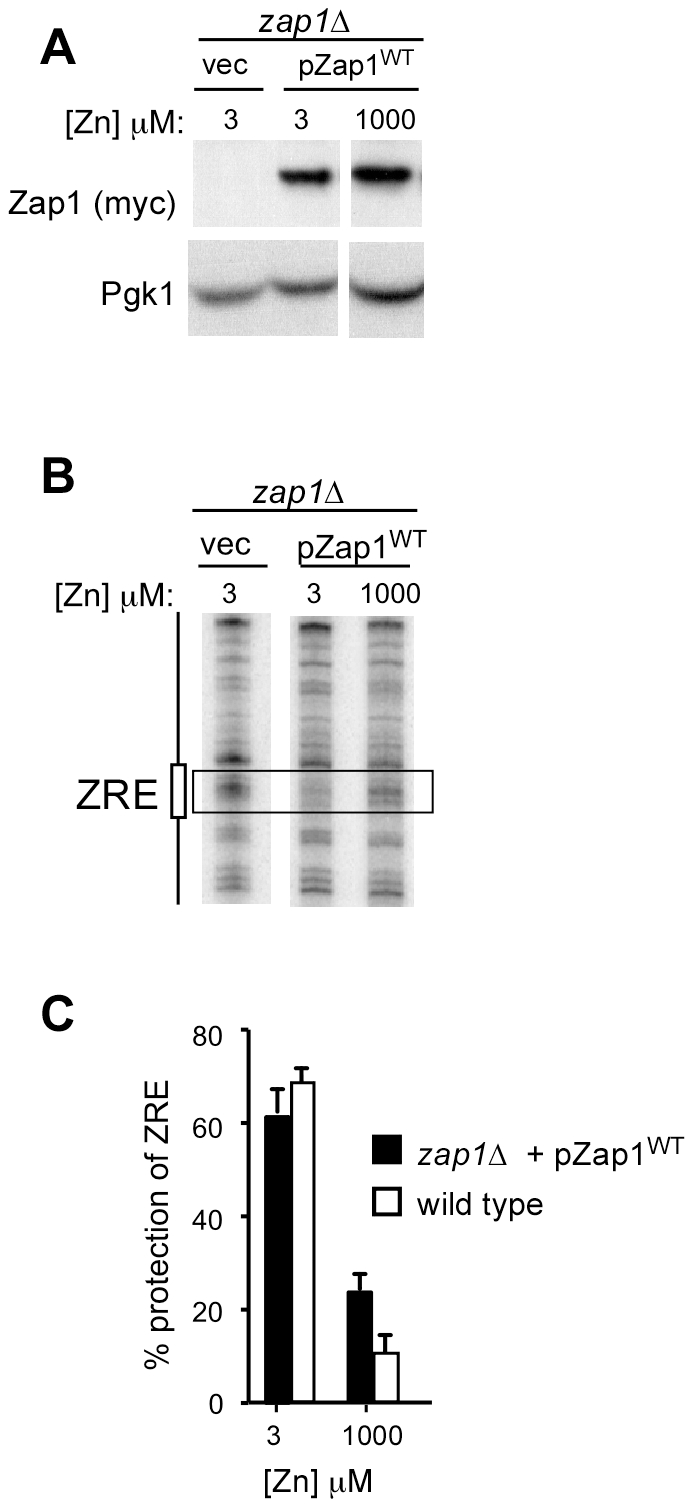
Zap1 DNA binding is controlled *in vivo* without autoregulation. **A**) ZHY6 *zap1Δ* mutant cells transformed with either pZap1^WT^ or the vector pYef2 were grown to exponential phase in LZM+3 µM and LZM+1000 µM ZnCl_2_. LZM contains 2% glucose as carbon source so expression of Zap1 from the *GAL1* promoter is at low levels similar to endogenous Zap1. Total protein extracts were prepared and subjected to immunoblot analysis using antibodies against Zap1 (myc) and Pgk1. **B**) ZHY6 *zap1Δ* cells transformed with the vector (pYef2) or pZap1^WT^ were grown to exponential phase in LZM+3 or 1000 µM zinc. Cells were than analyzed by *in vivo* DMS footprinting. The *box* indicates the position of the ZRE and the bands used for quantification of protection. **C**) The experiment shown in Figure 3B was repeated a total of six times and quantified as described in the Materials and Methods. The mean percent protection levels are shown and the error bars indicate 1 S.D. Data for wild-type cells from Figure 2B are shown again here for comparison.

To determine the contribution of autoregulation to controlling Zap1 binding, we examined ZRE occupancy using glucose-grown cells expressing Zap1 at a low constitutive level from the *GAL1* promoter. An epitope-tagged allele was used in which six myc epitopes were fused to the N-terminus of Zap1 to facilitate immunoblot detection. Notably, myc-Zap1 expressed from the *GAL1* promoter in glucose fully complemented a *zap1Δ* mutant for *ZRT1-lacZ* expression (**Figure 1A**). Immunoblotting confirmed that myc-Zap1 protein levels were similar in low and high zinc (**Figure 3A**). Using *in vivo* DMS footprinting, we found that protection of the *ZRT1* ZRE from methylation was apparent in zinc-limited cells and this protection decreased in high zinc (**Figure 3B**). To ensure the reproducibility of this result, we performed this experiment six times, quantified the intensities of the protected ZRE bands. These results are presented in **Figure 3C** alongside the wild type data from **Figure 2**. Zinc-regulated promoter occupancy was clearly observed in cells expressing Zap1 at a low constitutive level without autoregulation. Thus, the differential ZRE occupancy observed in wild-type cells is not due solely to changes in Zap1 protein level.

**Figure 4:**
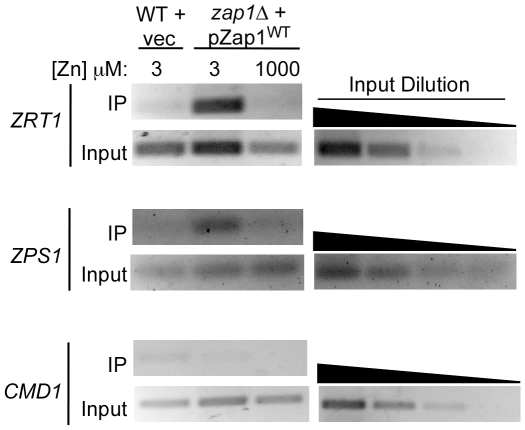
Confirmation of regulated Zap1 ZRE occupancy by chromatin immunoprecipitation. ZHY6 *zap1Δ* cells transformed with pZap1^WT^ were grown to exponential phase in LZM+3 µM or 1000 µM ZnCl_2_. Wild-type (DY1457) cells transformed with pYef2 vector were used as a negative control. Cells were then harvested and chromatin immunoprecipitation was performed using primers flanking the ZREs in *ZRT1* and *ZPS1*. Primers specific for the promoter region of *CMD1* were used as a negative control. Shown inputs are 1000-fold dilutions of whole cell extracts, and 10-fold serial dilutions of representative samples were also PCR amplified to confirm the quantitative nature of the assay.

To confirm these results using a different method, we used chromatin immunoprecipitation to assess ZRE occupancy *in vivo*. Again, myc-tagged Zap1 was expressed at a low level from the *GAL1* promoter in glucose-grown cells. When myc-Zap1 was immunoprecipitated from cross-linked chromatin, ZRE-containing promoter regions of *ZRT1* and *ZPS1* co-immunoprecipitated in samples from low zinc cells (**Figure 4**). Co-immunoprecipitation of these promoters was greatly reduced in high zinc cells. The *CMD1* promoter was used as a negative control and was not immunoprecipitated from either low- or high-zinc grown cells. These results confirm that Zap1 ZRE occupancy is zinc regulated in the absence of transcriptional autoregulation.

### Zinc regulation of ZRE occupancy occurs without changes in nuclear Zap1 localization

**Figure 5:**
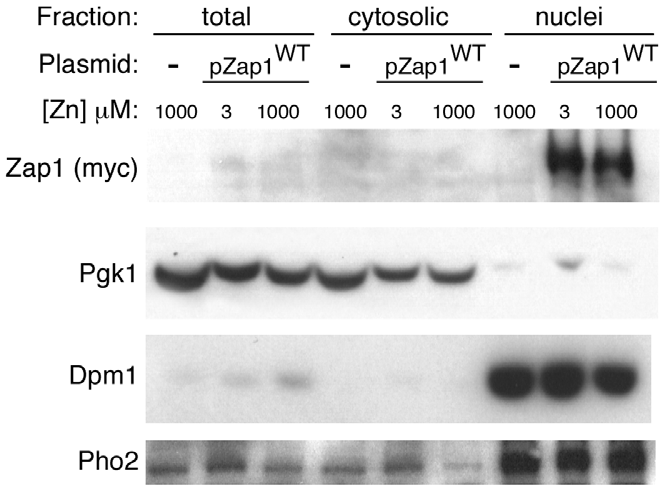
The nuclear localization of Zap1 is not affected by zinc. Protease-deficient BJ2168 cells transformed with pZap1^WT^ were grown to exponential phase in LZM supplemented with 3 µM or 1000 µM ZnCl_2_. BJ2168 cells lacking pZap1^WT^ were also grown in LZM supplemented with 1000 µM ZnCl_2_. Total cell homogenates were separated into cytosolic and nuclear fractions as described in Materials and Methods. Equal amounts of protein from each sample (10 µg protein/lane) were assayed by immunoblotting using antibodies against Zap1 (myc), Pgk1, Dpm1, and Pho2.

We previously demonstrated that Zap1's nuclear localization does not change in response to zinc status but those experiments were performed with cells overexpressing Zap1 to allow its detection by immunofluorescence microscopy [19]. Such overexpression conditions could potentially override zinc regulation of Zap1 subcellular distribution. Therefore, to examine Zap1 distribution at its normal low level of expression, we used immunoblotting to assay Zap1 levels in total lysates, cytosol, and isolated nuclei. Zap1 was expressed at a low constitutive level from the *GAL1* promoter in cells grown in glucose with low and high zinc. As shown in **Figure 5**, Zap1 levels were low in total lysates and cytosol but were highly enriched in nuclei regardless of zinc status. Pgk1 was used as a cytosolic protein marker and Dpm1 and Pho2 were markers for nuclei. Their distributions in these fractions confirmed our nuclear isolation method. These results demonstrate that Zap1 nuclear localization is not regulated by zinc status. Therefore, our DMS footprinting and chromatin immunoprecipitation results (**Figures 2**, **3**, and **4**) suggested that Zap1's DNA binding activity per se is regulated by zinc.

### Zinc regulation of ZRE binding maps to the Zap1 DNA binding domain

**Figure 6:**
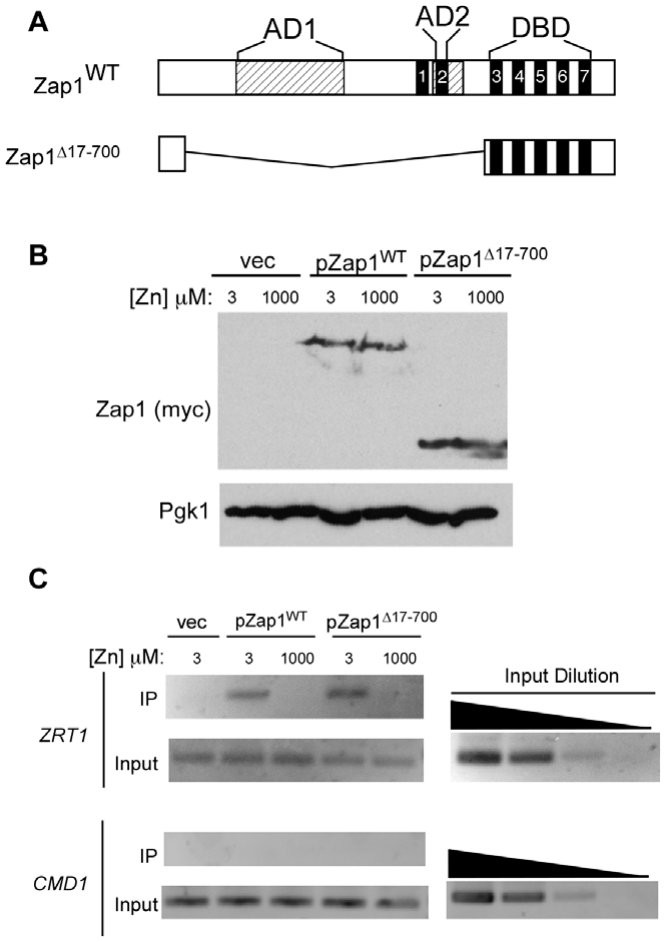
Zinc regulation of Zap1 ZRE binding maps to the DNA binding domain. **A**) Diagram of wild-type Zap1 and Zap1^Δ17-700^. The latter allele retains only a small part of the N-terminus of the protein and the intact DNA binding domain. The *hatched* boxes indicate the activation domains and the *black* boxes numbered 1–7 denote the zinc fingers. **B**) ZHY6 *zap1Δ* cells transformed with pPGK-ZRT1, and either pZap1^WT^, pZap1^Δ17-700^, or the pYef2 empty vector were grown to exponential phase in LZM supplemented with 3 µM or 1000 µM ZnCl_2_. pPGK-ZRT1 expresses the high affinity *ZRT1* zinc transporter from the *PGK1* promoter allowing a cell with no functional Zap1 to grow well in low zinc. Total protein extracts were prepared and subjected to immunoblot analysis using antibodies against Zap1 (myc) and Pgk1. **C**) The same cells as described in panel B were grown to exponential phase and chromatin immunoprecipitation analysis was performed using primers flanking the ZRE in *ZRT1*. Primers specific for the promoter region of *CMD1* were used as a negative control. The shown inputs are 1000-fold dilutions of whole cell extracts and 10-fold serial dilutions of representative samples are included to confirm the quantitative nature of the assay.

Zinc regulation of the GAD-Zap1^DBD^ fusion protein [19] suggested that control of ZRE occupancy maps to the DNA binding domain alone. To assess this hypothesis directly, we tested whether a Zap1 truncate containing only the DNA binding domain showed zinc-regulated ZRE occupancy *in vivo*. For this purpose, we used a myc-tagged deletion mutant in which amino acids 17-700 of Zap1 were deleted (**Figure 6A**). When expressed from the *GAL1* promoter at low levels, Zap1^Δ17-700^ accumulated to similar levels regardless of zinc status (**Figure 6B**). Chromatin immunoprecipitation indicated that *in vivo* ZRE occupancy of this truncated Zap1 protein was still zinc regulated (**Figure 6C**). These results indicate that this property maps to the Zap1 DNA binding domain alone and does not require other regions of the Zap1 protein.

### The contribution of Zap1 DNA binding control to Zap1 function and zinc homeostasis

**Figure 7:**
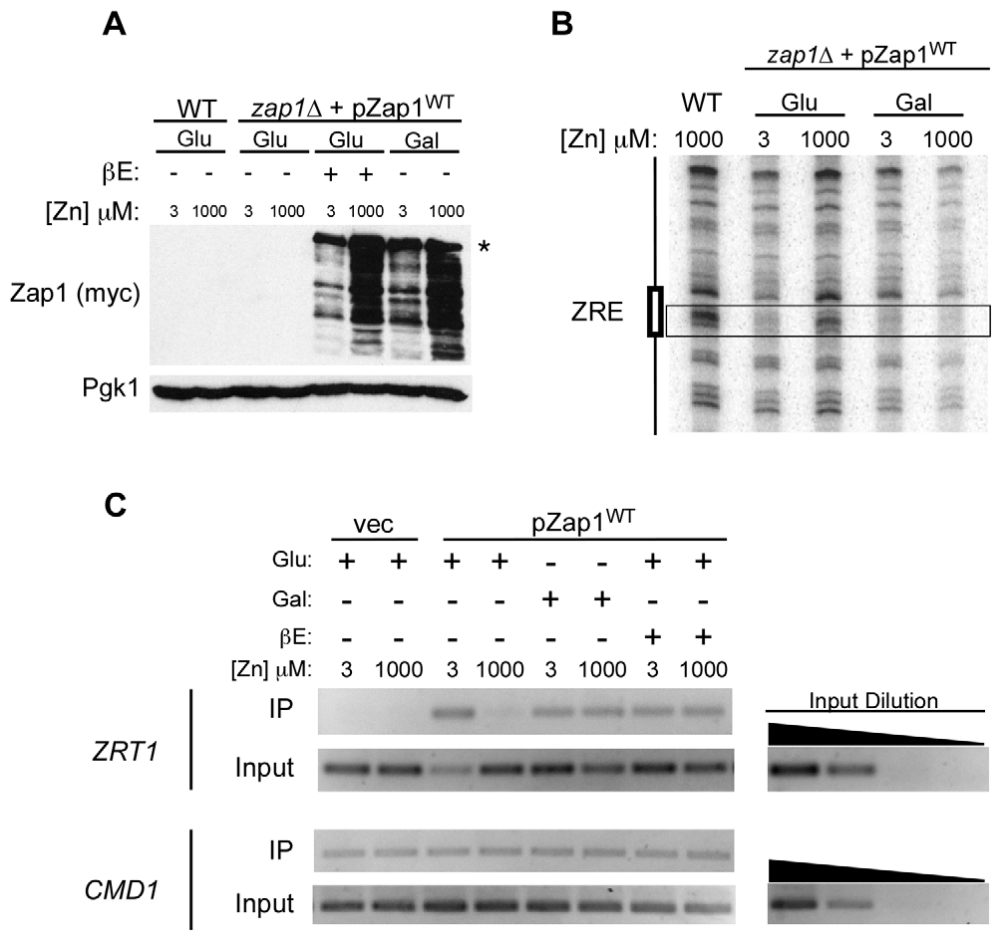
Overexpressing Zap1 protein overrides zinc regulation of DNA binding activity. **A**) Wild-type (DY1457) or ZHY6 *zap1Δ* cells transformed with pGEV and pZap1^WT^ were grown to exponential phase in LZM supplemented with 3 µM or 1000 µM ZnCl_2_. For low-level Zap1 expression, 2% glucose (Glu) was used as the carbon source. pGEV encodes a hybrid activator protein that contains the Gal4 DNA binding domain, the VP16 activation domain, and the hormone-response domain of the human estrogen receptor. For high-level expression, cells were grown in either 2% galactose (Gal) or glucose +1 µM β-estradiol (βE). Total protein extracts were prepared and subjected to immunoblot analysis using antibodies against Zap1 (myc) and Pgk1. The *asterisk* marks full-length myc-Zap1; the lower bands likely represent proteolytic fragments. **B**) Wild-type (DY1457) or ZHY6 *zap1Δ* cells transformed with pZap1^WT^ were grown to exponential phase in LZM supplemented with 3 µM or 1000 µM ZnCl_2_. For low-level Zap1 expression, 2% glucose (Glu) was used as the carbon source. To induce high Zap1 expression, 2% galactose (Gal) was used. *In vivo* DMS footprinting analysis was then performed. The *box* marks the position of the ZRE. **C**) ZHY6 *zap1Δ* cells transformed with either pZap1^WT^ or pYef2 vector were grown to exponential phase in LZM supplemented with 3 µM or 1000 µM ZnCl_2_. For low-level Zap1 expression, 2% glucose (Glu) was used as the carbon source. For high-level Zap1 expression, cells were either grown in the presence of 2% galactose (Gal) or glucose+1 µM β-estradiol (βE). Chromatin immunoprecipitation analysis was performed using primers flanking the ZRE of the *ZRT1* promoter. Primers specific to the promoter region of *CMD1* were used as a negative control. The shown inputs represent 1000-fold dilutions of whole cell extracts and 10-fold serial dilutions of representative samples are included to confirm the quantitative nature of the assay.

In the course of these studies, we found that the control of DNA binding activity was sensitive to the level of Zap1 expression. When *zap1Δ* cells bearing the *GAL1*-driven *ZAP1* gene were grown in galactose or overexpression was achieved using the GEV hybrid activator protein and high levels of the β-estradiol inducer, Zap1 protein accumulated to levels much higher than were observed in uninduced, glucose-grown cells (**Figure 7A**). The higher level of Zap1 accumulation in zinc-replete cells overexpressing the protein is likely an artifact of the expression system used and has been previously observed [16]. Zap1 accumulation in glucose-grown cells was below the level of detection on this blot. The multiple bands observed in samples from Zap1-overexpressing cells are likely due to some proteolysis of the protein. When Zap1 was expressed at these high levels and ZRE occupancy was assayed by *in vivo* DMS footprinting, the *ZRT1* ZRE was protected from methylation regardless of zinc concentration indicating constitutive Zap1 binding (**Figure 7B**). This conclusion was confirmed by chromatin immunoprecipitation of Zap1 on the *ZRT1* promoter in both high and low zinc (**Figure 7C**). Thus, high-level Zap1 expression can overwhelm zinc-responsive regulation of DNA binding activity.

**Figure 8:**
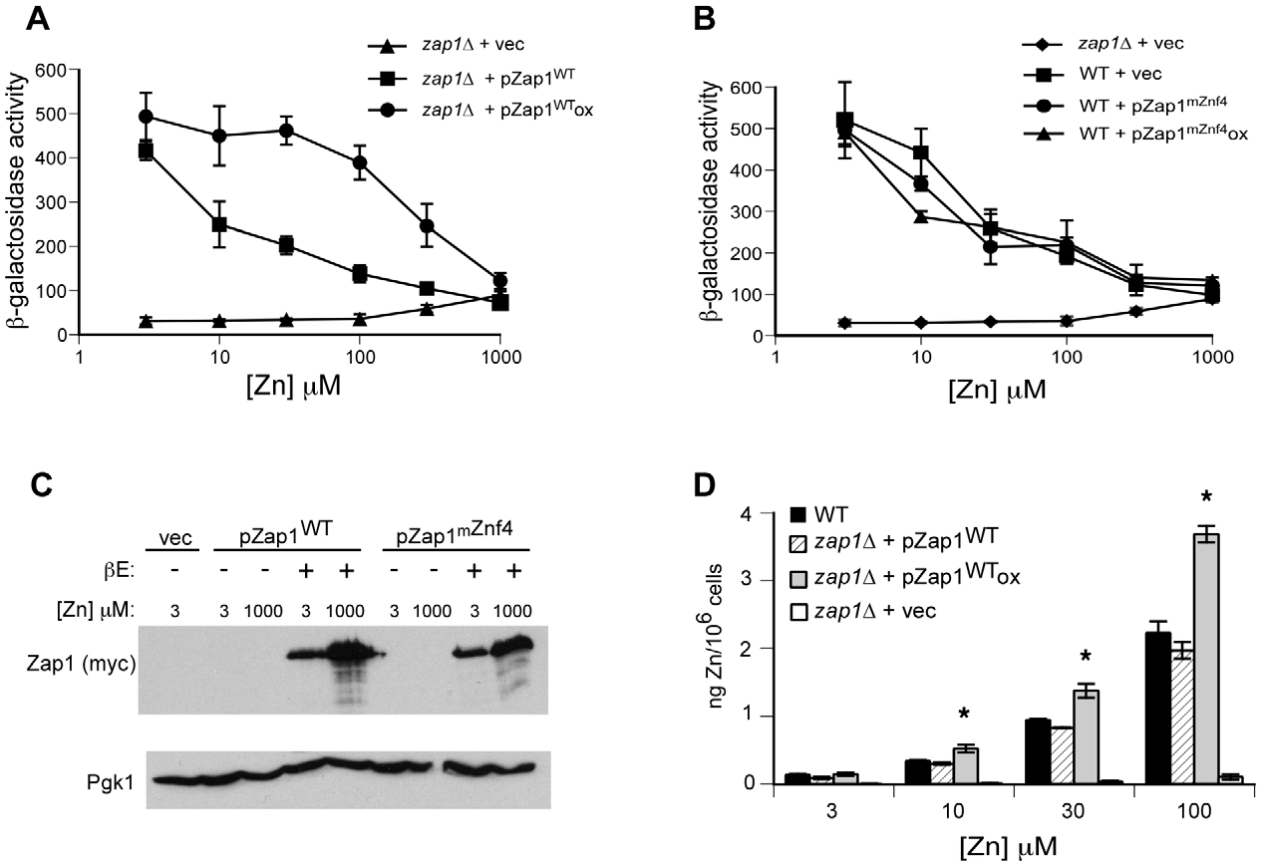
Overexpressing Zap1 disrupts gene regulation and zinc homeostasis. **A**) ZHY6 *zap1Δ* cells transformed with pGEV, the pGI-1 *ZRT1-lacZ* reporter and either pZap1^WT^ or pYef2 vector were grown to exponential phase in LZM supplemented with the indicated concentration of ZnCl_2_. To induce high Zap1 expression, cells were treated with 1 µM β-estradiol (ox). Cells were then harvested and β-galactosidase assays were performed. **B**) Wild-type DY1457 cells transformed with pGEV, the pGI-1 *ZRT1-lacZ* reporter, and either pYef2 or pZap1^mZnf4^ were grown to exponential phase in LZM supplemented with the indicated concentration of ZnCl_2_. To induce high Zap1^mZnf4^ levels, cells were treated with 1 µM β-estradiol (ox). Cells were then harvested and β-galactosidase assays were performed. The values shown in panels A and B are the means of three independent cultures, and the error bars equal ±1 S.D. **C**) Protein extracts were generated from the samples described in panels A and B and subjected to immunoblot analysis using antibodies against Zap1 (myc) and Pgk1. **D**) Wild-type DY1457 cells and ZHY6 *zap1Δ* cells transformed with pGEV and either the pYef2 vector or pZap1^WT^ were inoculated at an A_600_ of 0.02 in LZM supplemented with the indicated concentration of ZnCl_2_ plus tracer amounts of ^65^ZnCl_2_. To induce high levels of Zap1^WT^ protein expression, cultures were treated with 1 µM β-estradiol (Zap1^WT^ox). Cultures were grown to an A_600_ of ∼0.75, after which zinc accumulation was measured. Shown are the means of three independent cultures and the error bars indicate ±1 S.D. The *asterisks* indicate a significant difference (*p*<0.05) of *zap1Δ* cells expressing Zap1^WT^ox relative to Zap1^WT^ controls as determined by 2-sided Student *t*-test.

This observation was useful because it allowed us to test the importance of DNA binding regulation to overall Zap1 zinc responsiveness. Zap1 was expressed in a *zap1Δ* mutant at low levels, such that DNA binding domain control was operable, and at high levels where binding was constitutive. When assayed for expression of the *ZRT1-lacZ* reporter, regulation by overexpressed Zap1 was clearly defective over a wide range of zinc levels and higher levels of zinc were required for *ZRT1-lacZ* expression to be inhibited (**Figure 8A**). One possible explanation for this effect is that the higher Zap1 protein levels in an overexpressing cell may bind significant amounts of zinc and consequently alter zinc homeostasis. However, we found that this is unlikely to be the case. Overexpression of a Zap1 mutant allele where key DNA binding residues (i.e. zinc finger α-helical ^-^1,3,6 residues) in zinc finger 4 were mutated to alanines [15] had no effect on the zinc responsiveness of wild-type Zap1 (**Figure 8B**). This mutant protein accumulated to the same level as the wild-type protein (**Figure 8C**) and is unable to bind DNA [15]. Because this mutation does not alter any zinc ligands of zinc finger 4, the mutant protein's ability to bind zinc there and at other sites in Zap1 is unlikely to be affected [23]. Therefore, we conclude that a high level of zinc binding by overexpressed Zap1 protein is not responsible for the altered transcriptional response observed in **Figure 8A**. In addition, these data indicate that high level expression of functional Zap1 activation domains also does not alter Zap1's zinc responsiveness.

These results show that regulation of Zap1 DNA binding is important for the zinc responsiveness of Zap1 transcriptional regulation. To assess its importance to zinc homeostasis, we assayed zinc accumulation in cells with or without DNA binding control. An effect on zinc accumulation would reflect misregulation of the zinc uptake transporters. Wild-type cells and *zap1Δ* cells expressing either low or high levels of Zap1 were grown in LZM supplemented with 3, 10, 30 and 100 µM Zn and tracer amounts of ^65^Zn to assess total zinc accumulation. After ∼5 generations of growth in these media, the cells were harvested, washed to remove surface-bound zinc, and assayed for accumulation. As shown in **Figure 8D**, cells overexpressing Zap1, and therefore lacking DNA binding control, accumulated higher levels of total zinc than did wild-type cells and *zap1Δ* cells expressing low levels of Zap1. These results support the hypothesis that DNA binding control is critical for maintaining zinc homeostasis in these cells.

## Discussion

In this report, we have shown that Zap1 DNA binding activity is regulated by zinc status. This conclusion is based on our observations that ZRE occupancy is altered in response to zinc without any changes in the total level of Zap1 or the nuclear localization of the protein. ZRE occupancy was assayed using both *in vivo* DMS footprinting and chromatin immunoprecipitation. One limitation of the footprinting method is that it does not definitively show that the protein protecting the ZRE is Zap1 and not some other transcription factor. However, several observations support the conclusion that the methylation protection observed is due to Zap1 binding. First, no protection was detected in a *zap1Δ* mutant and protection was constitutive in a Zap1-overexpressing strain. Second, *in vitro* studies have shown that purified Zap1 binds to ZREs with high sequence specificity and affinity [14]. Electrophoretic mobility shift assays (EMSAs) using protein extracts prepared from cells indicated that Zap1 in these crude preparations binds to the ZRE (C. Srinivasan and D. Winge, unpublished). Specifically, protein-ZRE complexes observed with extracts from cells expressing myc-Zap1 could be super-shifted with anti-myc antibody. Finally, and most importantly, the DMS footprinting results were confirmed using an independent method, chromatin immunoprecipitation, which allowed us to directly show the regulated association of Zap1 with ZREs *in vivo*.

With the addition of DNA binding control, we now know that Zap1 activity is regulated by zinc via multiple mechanisms. Two of these mechanisms involve the regulation of Zap1's two activation domains. Our previous studies showed that zinc controls AD1 and AD2 independently of each other. This regulation likely occurs through the binding of zinc directly to residues within and flanking these activation domains. We hypothesize that zinc binding to these domains folds them into inactive conformations that are incapable of recruiting coactivators to Zap1 target promoters. We have also shown in previous studies that *ZAP1* gene expression is under positive transcriptional autoregulation. Here we confirm that this transcriptional control does indeed alter Zap1 protein levels.

Having so many different mechanisms of zinc responsiveness raises the issue of what each of these mechanisms contributes to Zap1 regulation and zinc homeostasis. With regard to transcriptional autoregulation, we found no obvious effect on regulation of a *ZRT1-lacZ* reporter when we eliminated that mechanism of control by expressing Zap1 at a constant low level from the *GAL1* promoter. However, we note that the *ZRT1* promoter contains four high affinity ZREs and therefore may be less sensitive to changes in Zap1 level than other promoters with lower affinity binding sites. This hypothesis is supported by our recent observation that Zap1 target genes with lower affinity ZREs tend to be induced only under the most severe zinc-limiting conditions when Zap1 protein levels are highest [9]. We therefore propose that transcriptional autoregulation of *ZAP1* expression may play a significant role on those promoters.

From our analysis of activation domain and DNA binding domain regulation, it is clear that these post-translational mechanisms are integrated to control the overall response of Zap1 to zinc. When comparing AD1 and AD2 function, we have found that AD1 is responsible for dictating the zinc dose response and induction kinetics of most Zap1 target genes [20]. In contrast, AD2 is required for full induction of only a few Zap1 targets. AD2 appears to play a more important role when zinc deficiency is combined with other stresses such as heat stress or carbon source limitation. Our studies of DNA binding regulation reported here suggest that this level of control also plays a major role in determining the zinc dose response of Zap1; when DNA binding was rendered constitutive by overexpression, Zap1 activity was much less responsive to zinc and zinc homeostasis was disrupted. Another potential role for Zap1 DNA binding regulation is that it may also contribute during transitions in zinc status. However, we found that constitutive Zap1 binding did not affect the rate at which *ZRT1* mRNA levels decreased following zinc treatment of zinc-limited cells, suggesting this is not the case (A. Frey, data not shown).

How might zinc control Zap1 DNA binding? We mapped the regulation of DNA binding activity to the Zap1 DNA binding domain alone. The five zinc fingers of the DNA binding domain are all high affinity structural zinc sites and these domains have metal bound even in zinc-limited cells. Our previous studies demonstrated that binding of zinc by each of the five fingers is required for ZRE binding [14]. One or more low affinity regulatory zinc-binding sites may also be present in the DNA binding domain. When intracellular zinc rises to sufficiently high levels, this regulatory site could then bind zinc and interfere with DNA binding, perhaps by promoting inhibitory finger-finger interactions. We have tested this model *in vitro* but these experiments did not support the hypothesis. Using both electrophoretic mobility shift assays and surface plasmon resonance analysis, we have determined that Zn^2+^ at concentrations as high as 10 µM does not inhibit ZRE binding by purified Zap1 (M. Evans-Galea and D. Winge, unpublished). This value is much higher than the nanomolar (or lower) levels of free zinc [6], [7], [8] found within cells. Higher concentrations of zinc did inhibit Zap1 ZRE binding *in vitro* but this effect was nonspecific and those levels of zinc also interfered with *in vitro* DNA binding of control proteins (Gal4, Swi5) that function well in zinc-replete cells. Thus, inhibition of DNA binding by zinc ions binding directly to the Zap1 DNA binding domain appears unlikely to be the mechanism that operates *in vivo*. An alternative model is that DNA binding activity is controlled by post-translational modification, such as phosphorylation. This form of DNA binding regulation has been demonstrated for other C_2_H_2_ zinc finger transcription factors, such as Adr1 [24]. Other work has shown that phosphorylation of the canonical TGEKP zinc finger linker region causes a strong reduction in DNA-binding affinity of C_2_H_2_ zinc finger proteins [25], [26]. Future studies will address further details regarding this mechanism of Zap1 DNA binding regulation.

## Materials and Methods

### Growth conditions

All yeast strains were grown in either YP medium supplemented with 2% glucose (YPD) or synthetic defined medium with 2% glucose and the appropriate auxotrophic supplements. Limiting zinc medium (LZM) was prepared as previously described [27] with either 2% glucose or 2% galactose as the carbon source and the indicated concentration of ZnCl_2_. LZM contains 1 mM EDTA and 20 mM citrate as metal buffers to limit zinc availability. It is important to note that even when supplemented with 1000–3000 µM zinc, LZM has lower free Zn^2+^ available to cells than is found in standard yeast culture media such as SD or YPD. Regulated expression from the *GAL1* promoter in glucose-containing media was achieved using the GEV system. The GEV protein contains the Gal4 DNA binding domain, the VP16 activation domain, and the hormone-response domain of the human estrogen receptor. Treatment of GEV-containing cells with β-estradiol results in increased expression of genes expressed from the *GAL1* promoter [28].

### Yeast strains and plasmids

The yeast strains used in this study were DY1457 (MATα *ade6 can1 his3 leu2 trp1 ura3*), ZHY6 (DY1457 *zap1*Δ::*TRP1*) [18], and BJ2168 (*MATa leu2 trp1 ura3 prb1 pep4 prc1 gal2*) [29]. The *ZRT1*-*lacZ* reporter construct used in this study was pGI-1 [22]. Plasmid pGI-1 contains the entire *ZRT1* promoter (−521 to the ATG start codon) fused to *lacZ*. The construction of pZap1^WT^ (previously referred to as pMyc-Zap1_1–880_), a plasmid containing an N-terminal myc-tagged *ZAP1* allele under the control of the *GAL1* promoter in the vector pYef2, was described previously [19]. pZap1^TC^ was generated as previously described (21). pZap1^Δ17-700^ was made by generating the corresponding open reading frame by overlap PCR and inserting the resulting fragment into BstX1-linearized pZap1^WT^ using homologous recombination. To create pPGK-ZRT1, a cloning strategy was used in which the *ZRC1* open reading frame in the construct pPGK-ZRC1 (C. W. MacDiarmid, unpublished) was replaced with *ZRT1* using homologous recombination. Primers were designed such that the 5′ primer contained 40 bp of homology to the *PGK1* promoter region and 20 bp of homology to the 5′ end of *ZRT1* and the 3′ primer contained 40 bp of homology to the *ZRC1* terminator and 20 bp of homology to the 3′ end of *ZRT1*. The resulting PCR product was co-transformed into DY1457 with *Bam*HI-digested pPGK-ZRC1 and transformants selected on SD media for the plasmid *URA3* marker. Plasmids were then rescued from DY1457 using standard procedures. The resulting construct containing *ZRT1* fused to the *PGK1* promoter and *ZRC1* terminator was confirmed by DNA sequencing.

### *In vivo* dimethyl sulfate footprinting

Thirty ml cultures were grown for 15–20 h to mid-exponential phase (A_600_ = 0.3–0.7) in LZM supplemented with the indicated amount of ZnCl_2_. Cells were treated with DMS (30 µl/30 ml of culture) for 7 min and then harvested by centrifugation. Isolation of genomic DNA and *in vivo* footprinting analysis were performed as previously described [30]. The oligonucleotide used for primer extension analysis (**Table 1**) hybridizes to the *ZRT1* promoter at positions −481 to −460 relative to the initiation codon. Quantification of band intensities was performed using a Storm 860 PhosphorImager (Molecular Dynamics) with ImageQuant software. Calculation of the fractional protection of residues was performed as follows [31]: Fractional protection (in percent) = 1−[(BI_ZRE_/BI_standard_)/(BI_ZRE_ in *zap1Δ*/BI_standard_ in *zap1Δ*)]×100 where BI_ZRE_ and BI_standard_ refer to the band intensities of the affected ZRE residues and of a control band elsewhere in the primer extension ladder that was unaffected by zinc or *ZAP1* genotype. Similar results were obtained when ZRE protection was quantified relative to several different control bands.

**Table 1:** Oligonucleotides used in this study.

Gene	Purpose	Sequence
*CMD1*	S1	5′-gggcaaaggcttctttgaattcagcaatttgttcttcggtggagcc-3′
*ZRT1*	S1	5′-ggccacacagattggtgtggttaacccatacgcaacacatagggcccatggccacctgatgcca-3′
*ZPS1*	S1	5′-ggcacccttggaaagcctccatcaattgctcaaagacacccatgacggtgaaaatactaccattaccgggttg-3′
*ZRT1*	*in vivo* footprinting	5′-gaacatggcgcaagtactta-3′
*CMD1*	ChIP (f′)	5′-cctccaatcttaccgaaga-3′
*CMD1*	ChIP (r′)	5′-gcgggagcaaaaaatcaca-3′
*ZRT1*	ChIP (f′)	5′-caatacacccgtactctcttgcctgt-3′
*ZRT1*	ChIP (r′)	5′-tgctctcaacctactttccatgac-3′
*ZPS1*	ChIP (f′)	5′-tcgacaatgacatggcggaag-3′
*ZPS1*	ChIP (r′)	5′-gaggttacattcttgtaagcag-3′

### Chromatin immunoprecipitation

Chromatin immunoprecipitation was performed as described [32]. Wild-type cells transformed with either the vector (pYef2) or a plasmid expressing a myc-tagged Zap1 protein (pZap1^WT^) were grown to an A_600_ ∼0.5 and then treated with 1% formaldehyde to cross-link protein-DNA complexes. The cross-linking reaction was quenched by adding 125 mM glycine. After two washes with ice-cold PBS, the cells were lysed with glass beads in buffer containing Complete Protease Inhibitor Cocktail (Roche), 1 mM PMSF, and 2 mM benzamidine. Following centrifugation for 10 minutes at 16,000× g, the supernatants were incubated with anti-myc antibody at 4°C overnight and immune complexes isolated with Protein A-Sepharose. The cross-links were reversed in TES and co-immunoprecipitation of specific promoter fragments with myc-Zap1 was assessed by PCR using primers flanking the *ZRT1* and *ZPS1* ZREs (**Table 1**). Primers specific to the *CMD1* promoter were used as a negative control. PCR products generated from 10-fold serially diluted input samples were used to confirm the quantitative nature of the analysis.

### Immunoblot analysis

Protein extracts for immunoblots were prepared by two different procedures. Subcellular fractionation analysis was performed as described previously [33] with the following modifications. Cells were grown to exponential phase in LZM supplemented with the indicated amount of ZnCl_2_. Spheroplasts generated by zymolyase (Zymo Research) digestion were resuspended in an 18% Ficoll buffer (20 mM potassium phosphate, pH 6.5, 0.5 mM MgCl_2_ and 18% Ficoll) and were disrupted using a Dounce homogenizer. Following removal of cell debris by centrifugation at 5000× g for 15 min, nuclei and other organelles were separated from the cytosolic fraction by centrifugation at 25,000× g for 30 min. The pellet was resuspended in 18% Ficoll buffer before being layered onto 35% Ficoll in the same buffer and the nuclei sedimented at 100,000× g for 90 minutes. All buffers during and after the disruption of spheroplasts contained 1 mM PMSF, 1 mM EDTA and the Complete Protease Inhibitor Cocktail (Roche). The protease-deficient strain BJ2168 was required for these experiments due to the extreme sensitivity of Zap1 to proteolytic degradation during sample preparation from wild-type cells. Total protein extracts for immunoblots were prepared by cell disruption and protein precipitation in the presence of trichloroacetic acid as described [34]. Immunoblots were performed essentially as described [35]. All proteins were separated by SDS-PAGE (7.5% acrylamide) and then transferred to nitrocellulose. Blots were probed with anti-Zap1 [15], anti-c-myc (monoclonal 9E10, Roche), or anti-Pgk1 (Molecular Probes) antibodies, washed and then incubated with either goat anti-mouse or goat anti-rabbit IgG antibodies coupled to horseradish peroxidase. Detection was by enhanced chemiluminescence (ECL; Amersham).

### S1 nuclease protection assays

RNA was extracted from cells grown to mid-log phase using hot acid phenol extraction. S1 analysis was performed as previously described [36]. Ten µg of total RNA was hybridized to a ^32^P end-labeled oligonucleotide probe before digestion by S1 nuclease and separation on an 8% polyacrylamide/8 M urea gel. Probes used are listed in **Table 1**. Band intensities were quantified by phosphorimager analysis.

### β-galactosidase assays

Cells were grown for 15–20 h to mid-exponential phase (A_600_ = 0.3–0.7) in LZM supplemented with the indicated amount of ZnCl_2_. β-galactosidase activity was measured as described [37] and activity units were calculated as follows: (ΔA_420_×1000)/(min×ml of culture×absorbance of the culture at 595 nm).

### Zinc accumulation assay

Cells were grown in LZM medium supplemented with the indicated concentration of ZnCl_2_ including ^65^ZnCl_2_ added in tracer amounts. The cells were grown to an A_600_ of ∼0.75, harvested by filtration on glass fiber filters, washed three times with ice cold SSW (1 mM EDTA, 20 mM sodium citrate pH 4.2), and then counted using a Wallac 1480 Wizard™gamma counter. Cell-associated ^65^Zn was then adjusted for specific activity and normalized by converting the culture A_600_ values to cell number with a standard curve.
